# Supplementary material for: Structural insights into CED-3 activation
Source: Life Sci Alliance. 2023 Jul 4;6(9):e202302056. doi: 10.26508/lsa.202302056 (PMC10320015; doi:10.26508/lsa.202302056)
Supplement: Supplementary file 1 [file LSA-2023-02056_TableS1.docx]

**Supplemental Table S1. Cryo-EM data collection and model statistics**

| Name | CED-4 | CED-4/CED-3  catalytic complex | CED-4/CED-3 _CARD complex | Holoenzyme |
| --- | --- | --- | --- | --- |
| **Data collection** |  |  |  |  |
| EM equipment | FEI Titan Krios | FEI Titan Krios | FEI Titan Krios | FEI Titan Krios |
| Voltage (kV) | 300 | 300 | 300 | 300 |
| Detector | Gatan K2 | Gatan K2 | Gatan K2 | Gatan K2 |
| Pixel size (Å) | 1.32 | 1.32 | 1.32 | 1.32 |
| Electron dose (e^-^/Å^2^) | 50 | 50 | 50 | 50 |
| Defocus range (μm) | 1.5~2.0 | 1.5~2.0 | 1.5~2.0 | 1.5~2.0 |
| **Reconstruction** |  |  |  |  |
| Software | RELION 3.0.7 | RELION 3.0.7 | RELION 3.0.7 | RELION 3.0.7 |
| Number of used particles | 125,140 | 23,339 | 67,312 | 115,378 |
| Symmetry | C1 | C7 (NOD)/  C1 (CARD) | C4 | C4 |
| Final resolution (Å) | 4.17/5.93 | 3.56/6.50 | 3.48/6.90 | 2.99/3.80 |
| **Model composition** |  |  |  |  |
| Protein residues | 2985 | 3,360 |  | 4,436 |
| ATP | 6 | 7 |  | 8 |
| Mg | 6 | 7 |  | 8 |
| **Validation** |  |  |  |  |
| R.m.s deviations |  |  |  |  |
| Bonds length (Å) | 0.01 | 0.009 |  | 0.012 |
| Bonds angle ( ̊) | 1.408 | 1.376 |  | 1.568 |
