# Supplementary material for: Structural insights into CED-3 activation
Source: Life Sci Alliance. 2023 Jul 4;6(9):e202302056. doi: 10.26508/lsa.202302056 (PMC10320015; doi:10.26508/lsa.202302056)
Supplement: Supplementary file 2 [file LSA-2023-02056_TableS2.docx]

**Supplemental Table S2. An overview of the oligomeric states of the four complexes under cryo-condition.**

| **Samples** | **Hexamer** | **Heptamer** | **Octamer** |
| --- | --- | --- | --- |
| CED-4 | 80.5% | 10.3% | 9.2% |
| CED-4/CED-3_CARD complex | 76.7% | - | 23.3%  (CED-3 binding) |
| CED-4/CED-3 catalytic complex | 43.1% | 40.5%  (CED-3 binding) | 16.4%  (CED-3 binding) |
| Holoenzyme | 47.9% | - | 52.1%  (CED-3 binding) |
